# Gene expression profiling of lymph node positive-negative metastasis of primary breast cancer in Saudi Arabian patients

**DOI:** 10.1186/1471-2164-15-S2-P55

**Published:** 2014-04-02

**Authors:** Adnan Merdad, Sajjad Karim, Hans-Juergen Schulten, Ashraf Dallol, Abdelbaset S Buhmeida, Fatima Al-Thubaity, Manar M Ata, Mamdooh A Gari, Adeel GA Chaudhary, Adel M Abuzenadah, Mohammed H Al-Qahtani

**Affiliations:** 1 Department of Surgery, King Abdulaziz University Hospital, King Abdulaziz University, Jeddah, Saudi Arabia; 2 Center of Excellence in Genomic Medicine Research, King Abdulaziz University, PO BOX: 80216 Jeddah 21589, Kingdom of Saudi Arabia

## Background

Breast cancer is the most frequent and most deadly cancer in females and incidence rates are increasing at an alarming rate in Saudi Arabia [1]. Studies have shown the potential of gene expression profiling in discovering biomarkers and molecular genetic signatures for breast cancer [2-4]. However, such discoveries and association of expression profiling with different type of Saudi Arabian breast cancer patients are largely unexplored.

## Materials and methods

We performed transcriptomic profiling of 42 surgically-resected breast tumors (n=23 lymph node positive; n=19 lymph node negative) using Affymetrix Gene 1.0 ST chip. Partek Genome Suit 6.4 and Ingenuity Pathway Analysis package were used for identification of differentially expressed genes, hierarchical clustering, gene ontology, pathway analysis and establishing clinical significance.

## Results

We identified 30 differentially expressed genes, including 10 up and 20 down-regulated in lymph node involved breast cancer using cut off of p value <0.05 and fold change >2. Ceruloplasmin (CP), immunoglobulin J polypeptide (IJP), cellular retinoic acid binding protein 1 (CRABP1), keratin 23 (KRT23), paraoxonase 3 (PON3), and integrin beta 6 (ITGB6) genes were up-regulated whereas genes like AF4/FMR2 family member 3 (AFF3), v-erb-a erythroblastic leukemia viral oncogene homolog 4 (ERBB4), anterior gradient homolog 2 (AGR2), serpin peptidase inhibitor clade A (SERPINA5), and POTE ankyrin domain family member E (POTEE) were downregulated. Transcriptomic signatures showed significant disruption in signaling pathways associating genes of the B-Cell development, Hematopoiesis from Pluripotent Stem Cells, Primary Immunodeficiency Signaling, Fatty Acid Biosynthesis Initiation II and LXR/RXR Activation.

## Conclusions

In the present study, we identified transcriptomic signature in Saudi breast cancer patients that is associated with lymph node metastasis. Our analysis reveals appropriate biological relevance and a number of molecular pathways that may serve as targets for novel therapeutics.

*Authors would like to acknowledge the KACST*, *Riyadh*, *Saudi Arabia* (*Project ID: 10BIO1073-03 and 10-BIO1258-03*) *for funding the research.*
